# The Role of Pathology in Small Renal Mass Laparoscopic Cryoablation

**DOI:** 10.1155/2012/539648

**Published:** 2012-03-19

**Authors:** B. W. Lagerveld, H. van Dekken, G. J. L. H. van Leenders, J. A. van der Zee

**Affiliations:** ^1^Department of Urology, St. Lucas Andreas Hospital, 1006 AE Amsterdam, The Netherlands; ^2^Department of Pathology, St. Lucas Andreas Hospital, 1006 AE Amsterdam, The Netherlands; ^3^Department of Pathology, Erasmus Medical Center, 3000 CA Rotterdam, The Netherlands

## Abstract

*Objective*. We evaluated histological outcome of intraoperative biopsies at laparoscopic renal mass cryoablation (LCA), prevalence of peritumoral fat tissue invasion, and risk of tract seeding. *Methods*. Patients were biopsied 3–5 times (16-gauge). Histology was analyzed by general pathologists and reviewed. Peritumoral fat was histologically examined. The trocar used for biopsy-guidance was examined by cytology. Records were studied for reporting tract metastasis. *Results*. 77 biopsied renal masses with mean ± SD diameter 30 ± 7.4 mm were histologically classified by primary and review pathology revealing 64 and 62 malignancies, 13 and 15 benign lesions, respectively. In 30/34, the fat covered a carcinoma but revealed no malignancy. Cytology showed no malignant cells but was inconclusive in 1 case. No tract metastasis occurred. *Conclusions*. The use of an intraoperative biopsy protocol provides histological diagnosis of all renal masses. No existence of peritumoral fat tissue invasion or tract seeding was found.

## 1. Introduction

The incidence of small renal tumors increases. More often these asymptomatic lesions are coincidentally found with cross-sectional imaging [1, 2]. Currently, the indication for surgical treatment of small renal masses is specifically based on radiographic imaging features, such as a consistent appearance and contrast enhancement [3]. However, tumor size and imaging alone are poor indicators for predicting the biological nature of the lesion [4–8]. Promising steps towards objectifying the relationship between radiological tumor anatomy and pathology were recently showed by Kutikov et al. [9], using the RENAL nephrometry score and the histology and grade of resected tumors.

The biological potential of small renal cell carcinomas (RCC) seems to be mild. This reflects in the relative merit cancer-specific survival of stage 1 disease [10]. However, it is stated that lesions of 3-4 cm diameter have a significant higher risk for high-grade and advanced-stage disease compared to smaller lesions [11]. Analysis of the Surveillance Epidemiology and End Results database suggested that stage 1 low-grade and high-grade RCC may appear in a more aggressive behaviour, in elderly patients, resulting in an increased cancer-specific mortality rate [10]. Today, urologists are increasingly faced with early-stage asymptomatic patients also having a renal mass ≤4 cm of potential malignancy. The majority is diagnosed in elderly patients bearing comorbidities that influence treatment decisions [12]. Especially, counselling this group of patients in the context of uncertain tumor pathology and biological potential remains a challenge.

Possible ways to avoid unnecessary, invasive treatment would be the use of diagnostic markers or to determine histology with image-guided core needle biopsies. However, the use of markers predicting the biological nature of renal masses ≤4 cm has not extensively been tested and validated. Although there is evidence that computed-tomography-guided percutaneous biopsies can help to distinguish between benign and malignant tissue, this method is not routinely used [13]. Hence, arguments such as sampling errors and possible track seeding are used to favor the risk of surgery in ignorance of histological diagnosis.

Currently, nephron-sparing surgery (NSS) is mandated for the invasive treatment of small renal masses. Among the various techniques qualifying the values of NSS, the partial nephrectomy is considered as the golden standard [14]. Laparoscopic partial nephrectomy is especially recognized for its challenging surgical skills and significant morbidity rate. Laparoscopic renal cryoablation (LCA) overcomes some of these shortcomings and is, therefore, gaining importance in the last decade.

The major limitation of LCA is the lack of tumor extirpative histology. So far, staging and follow-up pattern are based solely on the histological findings obtained by biopsy. Biopsies are performed image-guided preoperative or laparoscopic-assisted intraoperatively. Neither way the accuracy of the biopsy can be verified. Furthermore, staging is incomplete because biopsy specimen is not eligible for studying capsular or perirenal fat invasion. The last would stage a tumor ≤4 cm up from pT1a to pT3a according to the definition of the 7th TNM classification 2010 and is considered as a poor prognostic factor.

Another limitation of LCA is the analysis of long-term data on the oncological outcome. This can only be done if the histological nature of the treated masses is identified. However, limited reports reveal their malignant or benign nature of LCA-treated lesions. Often they show a significant percentage of benign and nondiagnostic biopsies. The RCC subtype and nuclear-grade differentiation are rarely mentioned. Furthermore, often the malignancy rate is lower than one would expect when comparing the rate to studies that report histology of renal masses obtained by extirpative surgery [8]. The role of materials and method of intraoperative biopsies needs to be studied.

For these above-mentioned limitations, the objective of this study was to evaluate the histological outcome of an intraoperative core needle biopsy protocol practiced at the time of LCA for small renal masses. The protocol should prevail in a small nondiagnostic rate and a malignancy rate that is comparable to series reporting on extirpation histology. We investigated also the existence of perinephric fat tissue invasion. Finally, the potential risk of tract seeding due to needle biopsy was analyzed.

## 2. Patients and Methods

From 2007 to August 2011, all patients with small renal masses treated with LCA were used for this study. All patients agreed with the intentions of the treatment. In a laparoscopic procedure, the renal mass was visually identified and confirmed with laparoscopic ultrasound.

The majority of the renal masses were exophytic. Critical point in the cryosurgical procedure was a staunch dissection of the tumor allowing identification of the lesion and its border. When the perirenal fat appeared to be denser and more adhesive to the tumor capsule, it was dissected and sampled for histological examination.

All renal masses were biopsied intraoperatively before cryoprobe placement. Laparoscopic ultrasound was used to avoid biopsies from cystic or necrotic parts in the lesions. For tissue sampling, we used a Magnum biopsy gun (Bard, Covington, Lousiana, USA), loaded with a disposable 16-gauge automatic side-cutting biopsy needle with a sample notch range of 19 mm. The device was fired through a trocar with the open end of the trocar close to the target. The biopsy needle was used percutaneously in case the distance through the trocar to the target was too long for reaching it properly. Trocar that was used for guidance of the biopsy needle was removed, rinsed, and submerged in saline. The saline was cytologically examined to determine the existence of tumor cells. Retrospectively, all patient records were studied for reporting metastasis in the tract of trocar, biopsy, and cryoprobe placement.

A standard of 3 biopsies were taken at different locations in the mass. The biopsy specimen was loaded off the needle on a glossy-coated paper surface. This method enabled to determine the value of all specimen obtained. More biopsies, with a maximum of 5, were taken when the value of 1 or more of the first 3 biopsies was considered to be limited at macroscopic inspection by the surgeon. Finally, all specimens were submerged in formalin. The biopsy and perirenal fat specimens were examined after normal hematoxylin and eosin (H&E) staining. If needed, on the biopsy specimen; immunohistochemical (IHC) staining (CD-10, CD-15, CD-117, CK-7, CK-20, Vimentin) was used to accept or to attain final diagnosis.

The first histology reports of the biopsies and perirenal fat were scored for the diagnosis indicating malignant or benign tissue. Tumor histology and subtype were reported according to the WHO 2004 classification (Bostwick and Cheng, 2008) [15]. The Fuhrman nuclear grade (1 to 4) was scored. To study the interobserver variability, two experienced uropathologists, ignorant for the goals of this study and the preliminary results of histology, reviewed the entire series of biopsies. The interobserver agreement on the lesions being malignant or not was calculated using Cohen kappa coefficient.

## 3. Results

A total of 81 LCA procedures on 80 small renal masses were carried out in 79 patients. Patient and tumor characteristics are shown in Table 1. One patient was treated for two lesions in the same kidney each in different operative sessions. 3 patients, of whom preoperative histological biopsies were proven to be malignant, were excluded. In 1 case, the tumor ablation was not completed as found at first evaluation using contrast computed-tomography (CT) and, thus, underwent a second LCA. The repeat biopsy was excluded from the study but revealed vital cells of the same clear cell carcinoma as found in the first procedure. In 3/81 (3.7%), the laparoscopic procedure was converted to an open cryoablative procedure. Extreme thick and adhesive perirenal fat prohibited a safe exposure without harming the kidney and the tumor and precluded an accurate targeting and, therefore, cryoablation. They remained included in the study because the biopsies were obtained according to the protocol. In total, 77 renal masses, with a mean ± SD diameter 30 ± 7.4 mm (range 15 to 50 mm), were intraoperatively biopsied. All tumors, as assessed by preoperative CT-scan and confirmed by laparoscopic ultrasound, were solid and round of form except for two cystic lesions that were classified as Bosniak III lesions.

All intraoperative biopsied renal masses were histologically classified. In total, 64 renal cortical cancers (83%) and 13 benign lesions (17%) were diagnosed. In 2 cases (2.6%), the biopsy specimens were diagnosed as renal cancer, but a subtype could not be specified. All malignancies showed no association with patient oncological history or transitional cell carcinoma. Both cystic lesions represented RCC. After review of the series, the blinded pathologists assessed 62 malignancies (80%) and 15 benign lesions (20%).  Table 2 shows the pathological characteristics from primary and review pathology.  Table 3 shows the interobserver agreement on a tumor being malignant or not. The relative observed agreement was (62 + 13)/77 = 0.974, and hypothetical probability of change agreement was {(64/77)×(62/77)}+{(13/77)×(15/77)} = 0.702. The Cohen kappa coefficient for this was (0.974 − 0.702)/(1 − 0.702) = 0.913. The reviewing pathologists agreed in 95% of the cases with the initial specification of the subtype of malignant or benign lesion. A discrepancy was noted in 4 cases as is specified in Table 4. In 47 cases where agreement was found between the observers considering the RCC subtype, both primary as review pathology assessed the nuclear grade. Agreement was found in 19 cases (40%). In 27 cases, the reviewing pathologists assessed the nuclear grade 1 to 2 grades higher and in 1 case (2%) 1 grade under.

Dense and adhesive peritumoral fat was specifically found in men 31/34 (91%). In 30/34 (88%), the fat covered a proven carcinoma (mean ± SD diameter 33 ± 7 mm, range 19–50 mm). Histology did not reveal malignancy in all cases. Cytology of the saline rinsed over the trocars was assessed in 33 biopsied tumors, of which 27 biopsied lesions were malignant. It showed a variety of cellular types such as blood, muscular, epithelial, and fat cells. In 1 case, cells could not be classified and were unsure of RCC origin. However, during followup (8 months), no sign of tract metastasis occurred. In all cases of RCC, with a mean followup period of 24 months (range 1–57 months), there was no existence of tract metastasis on physical examination or contrast CT-scan.

## 4. Discussion

The role of ordinary image-guided biopsies of a renal mass prior to intervention or during followup was not within the scope of this study. Within the context of that almost 20% of renal masses <4 cm will be benign [8, 16], it is not just that patients remain refrained from a final diagnosis after surgical treatment indented for a suspected malignancy. Besides the psychological importance for patients, several arguments support the need for an improved indexation of the biological nature of LCA-treated tumors. Although this method is currently a well-accepted treatment option, large series of long-term data are insufficient and final histology is often not reported. So far, the majority of the literature has focused more on the technical success based on follow-up imaging rather than combining it with histological diagnosis. This implicates that the clinical efficacy and success rate of cryosurgery as focal treatment of RCC cannot be objectified. Furthermore, the necessity and intensity of follow up using contrast radiographic imaging cannot be adjusted. So far, a risk-adapted followup has not been introduced in the literature. Unfortunately, there is no consensus on the follow-up of ablated small renal masses. This study shows a high interobserver agreement on the histological findings. Therefore, we consider that pathology findings of intraoperatively obtained biopsies can be used for adjustments of the routine followup.

This study is limited by a relatively small sample size, but it suggests that histological diagnosis can be obtained performing intraoperative core needle biopsies of a small renal mass prior to LCA. Sample errors are expected to be rare when biopsies are performed under direct laparoscopic vision and ultrasound guidance. In all cases, the biopsy strategy provided in enough tissue capacity for normal H&E and occasional IHC staining and resulted in a histological diagnosis. Furthermore, this study shows that there was no significant difference between the primary pathology and the review pathology by general pathologists and uropathologist, respectively.

Studies of LCA series that, also reported histology, showed a variation in malignancy percentage, normal renal tissue percentage, unclassified diagnosis rate, and benign tissue percentage [17–22]. The malignancy rate of 80% found in this study was higher compared to other LCA series. The malignancy rate of this study was in line with the expected malignancy rate reported in studies of small renal masses treated with extirpative surgery [8, 16]. Factors such as patient selection, tumor size, age, and biopsy quality may affect the undiagnosed rate and malignancy rate. The influence of patient selection will not be discussed.

It is reported that malignancy rate appears to be related to renal mass diameter [4, 6–8, 20, 23]. In this study, we noted that the mean renal mass diameter of 30 mm was larger compared to much other histology reporting LCA series in the literature (range 24–29 mm) [17–22], which may explain the higher malignancy rate. In this study, after primary pathology assessment, we found an increase of malignancy rate from 79% to 87% for lesions ≤3 cm and >3 cm, respectively. In general, patients with a maximal tumor diameter of 4 cm are offered cryoablation. However, at our referral centre for small renal masses, sometimes patients are presented with a renal mass >4 cm diameter mandating minimally invasive and nephron-sparing treatment. When all lesions >4 cm (*n* = 7) were excluded from this study, the malignancy rate was 80%. However, the mean ± SD tumor diameter remained 29 ± 5.9 mm, which may explain the higher malignancy rate.

Also, patient's age at the time of surgery can alter the malignancy rate [24]. It is stated that younger patients have a higher probability of bearing a malignancy. The average age in this study was 65,9 years, which was equivalent to most other series and, thus, other factor(s) than age must have caused the higher malignancy rate [17–19, 22]. However, comparing the data to Lehman et al. [20], we noticed that in both cohorts (≤3 cm and >3 cm) the mean age in this study was almost 10 years younger. Therefore, the lower malignancy rates (46.7% and 66.7%, resp.) found by Lehman et al. [20] at least need to be corrected for the factor age. Hence, for the other series another component must have caused the difference.

Several studies discussed the relationship between the amount of biopsy specimen and diagnosis rate [25, 26]. Johnson et al. [25] analyzed the results of sonographically guided renal mass biopsy and showed that using an 18-gauge core needle revealed more reliable diagnostic results compared to using a 20-gauge core needle. Kramer et al. [26] showed clearly that by increasing the number of intraoperative biopsy cores from 1 to 3 at LCA, the malignancy rate increased from 60% to 71%, whereas the nondiagnostic rate decreased from 17% to 5%. The protocol of minimal 3 biopsies using a 16-gauge needle with a sample notch range of 19 mm provided an excellent tissue sample size, which to our opinion was related to our high diagnosis rate.

This study is limited by the absence of histological confirmation because all tumors were ablated and not extirpated. Consequently, the false negative or positive rate and nuclear up- or downgrading of malignancies is unknown. The accuracy of the histology can only be examined in a prospective study comparing intraoperative biopsies to the histology of the extirpated lesion. We are not aware of a study with this design. Schmidbauer et al. [13] reported on the diagnostic accuracy of preoperative biopsies. They compared the histology of 2 or 3 18-gauge core needle CT-guided preoperative biopsies to that of the final extirpation specimen. The sensitivity for RCC detection was 93.5%. 2 sets of biopsies (3%) missed the target and were false negative showing normal renal parenchyma, whereas final histology revealed clear cell carcinoma. In a MEDLINE search, Volpe et al. [27] found that, at centers with expertise, an accurate diagnosis for malignancy was obtained in more than 90% of the percutaneous renal mass biopsies. Objectively, there is no reason to suppose that intraoperative biopsies are less accurate than percutaneous biopsies. Although some malignant subtypes are limited and difficult to discern or remain unclassified, this is not expected to account for a nondiagnostic rate >10% [28]. These data support that if biopsies are targeted appropriately and enough qualitative tissue is sampled an accurate histological diagnosis can be assessed.

Subtype, stage, and grade may help to predict small renal cancer-specific survival [4, 6, 8, 11]. Biopsy histology may require in a subtype diagnosis, but true staging remains incomplete, which is a limitation. Assessment of peritumoral fat invasion of small renal cancers would be helpful. So far, this study showed no invasion of peritumoral fat, but the data was biased by patient selection. However, in partial nephrectomy also tissue extirpation including perirenal fat is not standard. We recognize that the sample size of this study was limited and will continue to collect more data.

Schmidbauer et al. [13] found a nuclear grade accuracy rate of 76% determined in percutaneous biopsy specimen. This study revealed an interobserver agreement of 39% for the nuclear grade. However, the accuracy cannot be studied by the lack of conclusive histology of extirpated tumors. Nuclear grade is a characteristic used in preoperative prognostic nomograms for partial nephrectomy [29]. When in future nomograms for LCA may be developed, the role of nuclear grade based on biopsy specimen needs to be discussed.

The overall estimated risk for tract seeding due to percutaneous biopsies is <0.01% [30]. To our knowledge, no track seeding in LCA has been reported. The limited data of this study supported that there is no increased risk for seeding in LCA alongside the tracks of trocars, percutaneous biopsy, and percutaneous cryoprobes. However, the discovery of cells of uncertain RCC-origin supports us to continue sampling data of trocar cytology to assess further the potential risk of seeding.

The focus of this paper was not on complications of intraoperative biopsies. Today, there is no study reporting complications of intraoperative renal biopsies. However, image-guided diagnostic biopsies have a low complication rate of which bleeding is most frequently reported. In our series, bleeding was minimal and could be taken care of during surgery. So far, in our series of renal mass LCA, we did not encounter a high rate of complications. The complication rate is 17% (Clavien-score 1-3a), which is comparable to the reported complication rates of renal mass LCA in the literature.

## 5. Conclusions

The intraoperative biopsy protocol for small renal masses provided histological diagnosis with a high-interobserver concordance for the RCC-subtype and avoided unnecessary nondiagnostic biopsies. The malignancy rate of 80% was comparable to series reporting on extirpative histology. No existence of peritumoral fat tissue invasion was found. Tract seeding in LCA did not exist. Further studies are needed to improve staging of small renal masses treated with LCA.

## Figures and Tables

**Table 1:** Patient and tumor characteristics.

Patients (*n*)	
Male	55
Female	24
Patient age (yr) (range)	65,9 (36–83)
Mean (SD yr)	68 (10.6)
Kidney (*n*)	
Right	46
Left	35
Indications (*n*)	
Solitary kidney	7
Tumor recurrence	1
2nd tumor ipsilateral kidney	1
Other	72
Tumors (*n*)	78
Tumor size (mm) (range)	30,6 (15–50)
Mean (SD mm)	30 (7.6)
LCA approach (*n*)	
Retroperitoneal	46
Transperitoneal	35
Conversion	3

**Table 2:** Pathological characteristics of 77 intraoperatively biopsied renal masses.

Histology	Primary pathology (*n*)	Review pathology (*n*)
Renal cell carcinoma		
Clear cell	43	41
Papillary	11	13
Chromophobe	8	8
Undefined	2	
Angiomyolipoma	3	3
Oncocytoma	10	12

**Table 3:** Interobserver agreement on renal masses diagnosed malignant or benign. Observer 1 represents the primary diagnosis by general pathologists. Observer 2 represents the diagnosis as determined by the blinded review pathologists.

		Observer 1		
		*benign*	*malignant*	Total observer 2
Observer 2	*benign*	13	2	15
	*malignant*	O	62	62
	Total observer 1	13	64	77

**Table 4:** Specifications of 4 subtype diagnoses that were differently scored by primary pathology and review pathology. RCC: renal cell carcinoma.

Primary diagnosis	Review uropathologists diagnosis
RCC not specified	oncocytoma
Clear cell RCC	oncocytoma
RCC not specified	papillary RCC
Clear cell RCC	papillary RCC

## References

[1] Chow WH, Devesa SS, Warren JL, Fraumeni JF (1999). Rising incidence of renal cell cancer in the United States. *Journal of the American Medical Association*.

[2] Cooperberg MR, Mallin K, Ritchey J, Villalta JD, Carroll PR, Kane CJ (2008). Decreasing size at diagnosis of stage 1 renal cell carcinoma: analysis from the National Cancer Data Base, 1993 to 2004. *Journal of Urology*.

[3] Silverman SG, Israel GM, Herts BR, Richie JP (2008). Management of the incidental renal mass1. *Radiology*.

[4] Frank I, Blute ML, Cheville JC, Lohse CM, Weaver AL, Zincke H (2003). Solid renal tumors: an analysis of pathological features related to tumor size. *Journal of Urology*.

[5] Klatte T, Patard JJ, de Martino M (2008). Tumor size does not predict risk of metastatic disease or prognosis of small renal cell Carcinomas. *Journal of Urology*.

[6] Hsu RM, Chan DY, Siegelman SS (2004). Small renal cell Carcinomas: correlation of size with tumor stage, nuclear grade, and histologic subtype. *American Journal of Roentgenology*.

[7] Remzi M, Katzenbeisser D, Waldert M (2007). Renal tumour size measured radiologically before surgery is an unreliable variable for predicting histopathological features: benign tumours are not necessarily small. *Brtish Journal of Urology International*.

[8] Van Poppel H, Joniau S (2007). Is surveillance an option for the treatment of small renal masses?. *European Urology*.

[9] Kutikov A, Smaldone MC, Egleston BL (2011). Anatomic features of enhancing renal masses predict malignant and high-grade pathology: a preoperative nomogram using the RENAL nephrometry score. *European Urology*.

[10] Sun M, Abdollah F, Bianchi M (2011). A stage-for-stage and grade-for-grade analysis of cancer-specific mortality rates in renal cell carcinoma according to age: a competing-risks regression analysis. *European Urology*.

[11] Remzi M, Özsoy M, Klingler HC (2006). Are small renal tumors harmless? Analysis of histopathological features according to tumors 4 Cm or less in diameter. *Journal of Urology*.

[12] Hollingsworth JM, Miller DC, Daignault S, Hollenbeck BK (2006). Rising incidence of small renal masses: a need to reassess treatment effect. *Journal of the National Cancer Institute*.

[13] Schmidbauer J, Remzi M, Memarsadeghi M (2008). Diagnostic accuracy of computed tomography-guided percutaneous biopsy of renal masses. *European Urology*.

[14] Campbell SC, Novick AC, Belldegrun A (2009). Guideline for management of the clinical T1 renal mass. *Journal of Urology*.

[15] MacLennan GT, Cheng I, Bostwick DG, Cheng I (2008). Neoplasms of the kidney. *Urological Surgical Pathology*.

[16] Pahernik S, Ziegler S, Roos F, Melchior SW, Thüroff JW (2007). Small renal tumors: correlation of clinical and pathological features with tumor size. *Journal of Urology*.

[17] Guazzoni G, Cestari A, Buffi N (2010). Oncologic results of laparoscopic renal cryoablation for clinical T1a tumors: 8 years of experience in a single institution. *Urology*.

[18] Schwartz BF, Rewcastle JC, Powell T, Whelan C, Manny T, Vestal JC (2006). Cryoablation of small peripheral renal masses: a retrospective analysis. *Urology*.

[19] Weight CJ, Kaouk JH, Hegarty NJ (2008). Correlation of radiographic imaging and histopathology following cryoablation and radio frequency ablation for renal tumors. *Journal of Urology*.

[20] Lehman DS, Hruby GW, Phillips CK, Mckiernan JM, Benson MC, Landman J (2008). First prize (tie)—Laparoscopic renal cryoablation: efficacy and complications for larger renal masses. *Journal of Endourology*.

[21] Barwari K, Beemster PWT, Hew MN, Wijkstra H, De La Rosette J, Laguna MP (2011). Are there parameters that predict a nondiagnostic biopsy outcome taken during laparoscopic-assisted cryoablation of small renal tumors?. *Journal of Endourology*.

[22] Klatte T, Grubmüller B, Waldert M, Weibl P, Remzi M (2011). Laparoscopic cryoablation versus partial nephrectomy for the treatment of small renal masses: systematic review and cumulative analysis of observational studies. *European Urology*.

[23] Thompson RH, Kurta JM, Kaag M (2009). Tumor size is associated With malignant potential in renal cell Carcinoma cases. *Journal of Urology*.

[24] Rendon RA, Jewett MAS (2006). Expectant management for the treatment of small renal masses. *Urologic Oncology*.

[25] Johnson PT, Nazarian LN, Feld RI (2001). Sonographically guided renal mass biopsy: indications and efficacy. *Journal of Ultrasound in Medicine*.

[26] Kramer BA, Whelan CM, Vestal JC, Schwartz BF (2009). Increasing the number of biopsy cores before renal cryoablation increases the diagnostic yield. *Journal of Endourology*.

[27] Volpe A, Kachura JR, Geddie WR (2007). Techniques, safety and accuracy of sampling of renal tumors by fine needle aspiration and core biopsy. *Journal of Urology*.

[28] Eble JN, Sauter G, Epstein JI, Sesterhenn IA (2004). *World Health Organization Classification of Tumours: Pathology and Genetics of Tumours of the Urinary System and Male Genital Organs*.

[29] Lane BR, Babineau D, Kattan MW (2007). A preoperative prognostic nomogram for solid enhancing renal tumors 7 cm or less amenable to partial nephrectomy. *Journal of Urology*.

[30] Herts BR, Baker ME (1995). The current role of percutaneous biopsy in the evaluation of renal masses. *Seminars in Urologic Oncology*.

